# Mitral valve disease in ankylosing spondylitis: an autoimmune disease manifestation? A case report

**DOI:** 10.1093/ehjcr/ytac322

**Published:** 2022-08-01

**Authors:** Sulayman El Mathari, Allard van der Wal, Hennie Raterman, Jolanda Kluin

**Affiliations:** Department of Cardiothoracic Surgery, Amsterdam University Medical Center, 1105 AZ Amsterdam, The Netherlands; Department of Pathology, Amsterdam University Medical Center, 1105 AZ Amsterdam, The Netherlands; Department of Rheumatology, North West Clinics, 1815 JD Alkmaar, The Netherlands; Department of Cardiothoracic Surgery, Amsterdam University Medical Center, 1105 AZ Amsterdam, The Netherlands

**Keywords:** Mitral valve disease, Mitral stenosis, Bechterew’s disease, Ankylosing spondylitis, Histopathology, Case report

## Abstract

**Background:**

Ankylosing spondylitis (AS) is a chronic inflammatory disease of primarily the joints of the spine. In the literature, AS is known to have cardiac manifestations. Most frequently, this is aortic regurgitation. However, in rare cases also mitral valve (MV) disease is observed in AS patients. The extent and mechanism of this involvement are still unclear. We aim to describe a histologically validated case report to add understanding on this topic.

**Case summary:**

We show the case of a 51-year-old male who suffered since his youth from back pain and uveitis, which was later diagnosed as AS. After a first presentation with combined heart valve disease, the patient recovered on cardiac medical therapy and biologic treatment for AS. Four years later, cardiac complaints worsened mainly due to severe MV stenosis. Surgical treatment was performed with histopathologic analysis of the excised MV validating involvement of AS.

**Discussion:**

Histopathologic analysis showed chronic fibro inflammatory thickening of the MV leaflets and subvalvular apparatus. These pathological features could fit with the inflammatory nature of AS. Since this is a rare case, the recognition of fibro inflammatory thickening leading to commissural fusion and stenosis may contribute to better understanding of heart valve disease in AS to create a base for better cardiac management in this specific patient group.

**Learning points**

- Mitral valve (MV) stenosis is a possible cardiac manifestation of ankylosing spondylitis (AS).
- The histopathology of the resected MV in the present study suggests an autoimmune rather than autoinflammatory origin of AS induced heart valve disease.
- Vigilance is required for heart valve disease in patients with AS.

## Introduction

Ankylosing spondylitis (AS), also known as Bechterew’s disease, is a chronic inflammatory disease of the spine and the most common type of axial spondylartritis (SpA). Axial spondylartritis primarily affects the axial and sacroiliac joints and occurs predominantly in male individuals. Extra spinal manifestations of AS include uveitis, inflammatory bowel disease, psoriatic skin lesion, kidney and cardiac involvement.^1^

Cardiac involvement in patients suffering from AS has been estimated to be 2–10%.^2,3^ The most frequently observed cardiac involvements include conduction disorders, aortic valve (AV) regurgitation, aortitis of the ascending aorta, and diastolic left ventricular (LV) dysfunction.^4–7^

The aetiology of AS is unknown and, as recently highlighted by Mauro *et al*. in Nature Reviews Rheumatology, there is an ongoing debate whether AS is an autoimmune or autoinflammatory disease.^8^ Autoinflammatory diseases are characterized by unprovoked inflammation induced by activation of the innate immune system in the absence of autoantibodies or autoantigen-specific T-cells. Whereas in contrast, autoimmune diseases are pathological conditions caused by an aberrant and prolonged adaptive immune response to a self-antigen. The review ends by stating: ‘Further research that finally enables us to determine whether AS is promoted by a prevalent autoinflammatory or autoimmune mechanism could have real-world implications, guiding the research agenda and contributing to understanding patient heterogeneity’.

We present a case of a 51-year-old man having AS with severe three valve disease most prominent in the mitral valve (MV). We aim to describe a case report to add understanding on the valvular pathology in AS, which might be helpful for future treatment strategies of the disease.

## Timeline

**Table:** 

Timeline	
1983–2017	Chronic lower back pain and recurring uveitis
2016 July	First cardiac presentation with mitral, aortic, and tricuspid valve (TV) regurgitation and a thickened aspect of the MV and its subvalvular apparatus, diagnosed with transthoracic echocardiography, transoesophageal echocardiography, and magnetic resonance imaging (MRI). Start cardiac medical therapy (angiotensin-converting enzyme-inhibitor and diuretics)
2016 December	Mitral regurgitation diminished and LV ejection fraction (LVEF) improved after starting cardiac medical therapy. Diagnosis AS and start biologic treatment with tumor necrosis factor-*α inhibitor adalimumab*
2017–20	Stable complaints with cardiac medical therapy and biologic treatment
2020 July	Increased cardiac complaints with dyspnoea on minimal exertion (NYHA Class III)
2020 December	Surgical mitral and AV replacement and TV repair
2021 February	Histopathologic analysis of excised MV validating as involvement

## Case presentation

**Figure 1:**
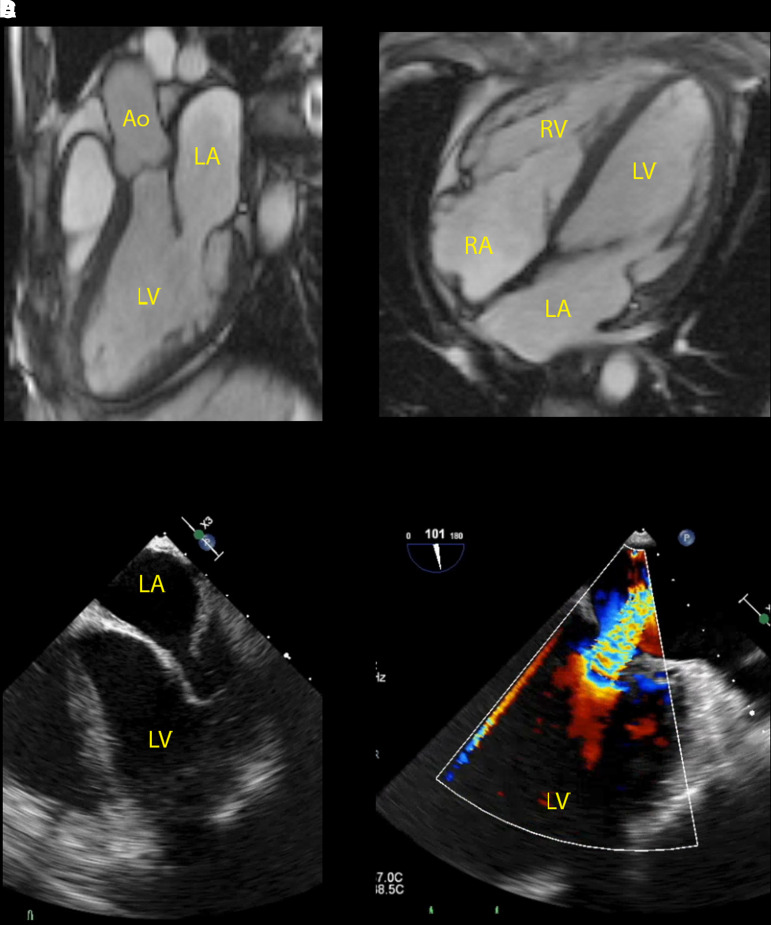
Imaging at first presentation showing severe mitral valve regurgitation with thickened mitral valve cusps and subvalvular apparatus on magnetic resonance imaging (*A* and *B*) and transoesophageal echocardiography (*C* and *D*). Ao, aorta; LA, left atrium; LV, left ventricle; RA, right atrium; RV, right ventricle.

A 51-year-old male presented 5 years ago for the first time with cardiac complaints. He was referred to our hospital with dyspnea, general malaise, and a Grade 4 systolic murmur combined with a Grade 2 diastolic murmur at physical examination. At lung auscultation, there were normal respiratory sounds without crackles and there was no peripheral oedema observed. Abdominal percussion sounds were normal and joints were not red or swollen. Electrocardiography and chest X-ray did not show any abnormalities. Transthoracic echocardiography (TTE) showed LV dilation, severe MV, and TV regurgitation, thickening of MV leaflets, and a reduced LVEF, whereupon transoesophageal echocardiography (TEE) and MRI were performed. Transoesophageal echocardiography and MRI confirmed LV dilation, severe MV and TV regurgitation, and moderate MV stenosis with a thickened aspect of the MV cusps and its subvalvular apparatus (*Figure 1* and *Video 1*). The mechanism of MV regurgitation seemed to be a combination of both degenerative (thickening of valve leaflets) and functional (LV dilation) valve disease, whereas the mechanism of TV regurgitation was functional (annular dilation). Furthermore, the AV showed mild regurgitation, LVEF was mildly reduced (41%), there were no signs of wall motion abnormalities and MRI showed delayed subendocardial enhancement in the mid inferior wall suggestive for myocardial oedema. Further analysis with coronary angiography and positron emission tomography-computed tomography scan excluded the possibility of endocarditis, myocarditis, and coronary disease. Lab results showed increased inflammation values C-reactive protein (CRP) 47 mg/L and erythrocyte sedimentation rate (ESR) 48 mm/h.

Besides cardiac complaints at presentation, the patient suffered since his youth from chronic lower back pain and recurring uveitis anterior. In earlier years, extensive examination with blood tests and spinal X-rays for these complaints were performed on suspicion of rheumatic disease, including rheumatic fever, but with negative results.

Due to these unexplained noncardiac complaints and suspicion of a link with the observed heart valve disease, further examination was performed. X-ray and MRI of the sacroiliac and low spine joints showed symmetric sacroiliitis. Following consultation of the rheumatologist resulted in the diagnosis AS with a human leucocyte antigen (HLA) B27-negative profile. According to European Alliance of Associations for Rheumatology recommendations and persistent high disease activity after non-steroidal anti-inflammatory drugs treatment with Naproxen (500 mg twice a day), adalimumab (TNF-α inhibitor) was initiated as biologic treatment.^9^ Disease activity was defined and monitored as complaints specific for AS and inflammation values (CRP and ESR).

Together with cardiac medical therapy [angiotensin-converting enzyme inhibitors (Fosinopril) and diuretics (Eplerenone)], both cardiac and musculoskeletal complaints diminished to an acceptable level. Because cardiac complaints and mitral regurgitation diminished and LVEF improved after starting cardiac medical therapy, the medical team remained expectative. The patient was discharged and remained under control in the outpatient clinic. In the following 4 years, the complaints remained stable with cardiac medical therapy and biological treatment, however, then cardiac complaints started to increase slowly again (see TTE findings during follow-up in *Table 1*).

**Table 1:** Findings at transthoracic echocardiography starting from first clinical admission through follow-up

Year	Left ventricular ejection fraction (%)	Mitral regurgitation (grade)	Tricuspid regurgitation (grade)	Aortic regurgitation (grade)
2016 July	41%	Grade 4	Grade 4	Grade 1
Start cardiac medical therapy (ACE-inhibitor and diureticts) and NSAIDS				
2016 December	50%	Grades 2–3	Grades 2–3	Grade 1
Start biologic treatment with adalimumab (TNF-α inhibitor)				
2017	50%	Grade 3	Grade 2	Grade 1
2018	64%	Grade 3	Grade 2	Grade 2
2019	55%	Grade 3	Grade 2	Grade 3
2020	43%	Grade 4	Grade 2	Grade 3
Surgical treatment with mitral and aortic valve replacement and tricuspid valve repair				

**Figure 2:**
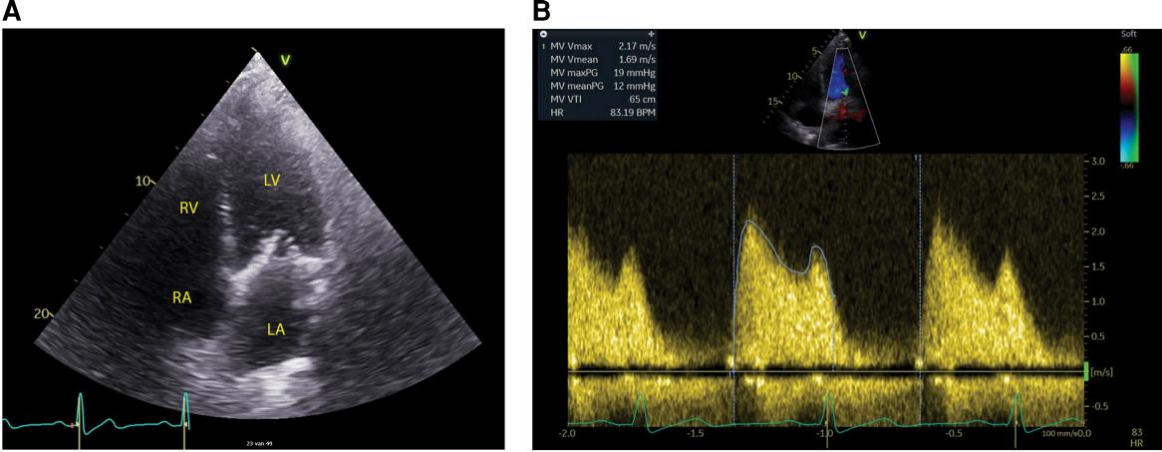
Preoperative transthoracic echocardiography four-chamber view (*A*) showing severely thickened mitral valve cusps and pressure assessment (*B*) of the mitral valve showing a mean pressure gradient of 12 mmHg and a maximum pressure gradient of 19 mmHg. PG, pressure gradient; LA, left atrium; LV, left ventricle; RA, right atrium; RV, right ventricle.

Four years after first presentation, the patient presented with progressive dyspnoea on minimal exertion. Transthoracic echocardiogram and TEE revealed increased thickening of MV leaflets causing severe mitral stenosis with a mean and maximum pressure gradient of, respectively, 12 and 19 mmHg (*Figure 2* and *Video 2*) and MV area was 2.1 cm^2^. This was combined with mitral, tricuspid, and AV regurgitation of, respectively, Grade 4, Grade 2, and Grade 3. Furthermore, left atrial size was 50.1 mm, MV diameter was 40 mm, and LVEF was 43%. The patient was treated surgically with MV replacement, AV replacement, and TV repair. The excised MV (*in toto*) and AV (in fragments) were preserved for histopathologic analysis. There were no relevant complications in the postoperative setting and the patient was discharged 7 days after surgery.

**Figure 3:**
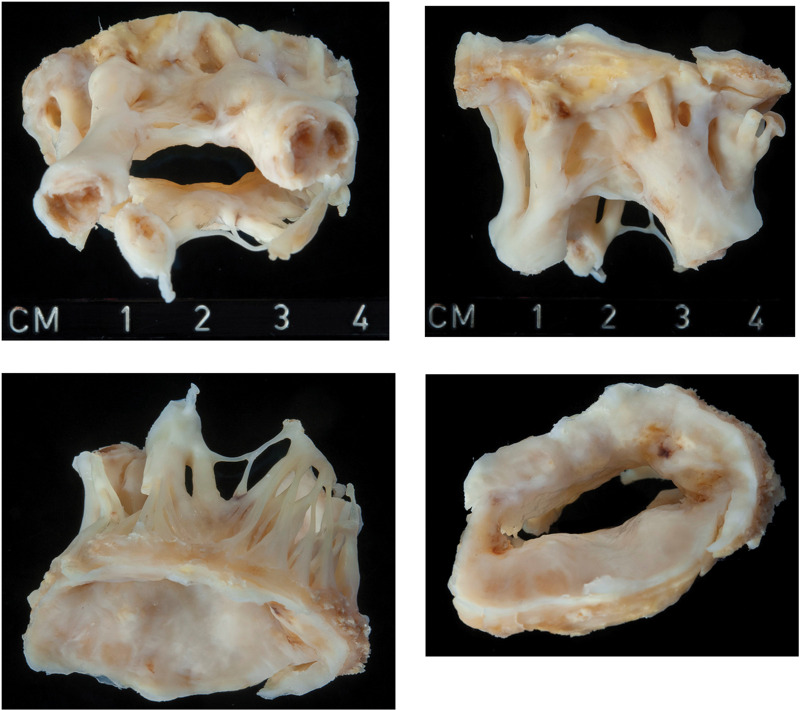
Macroscopic appearance of mitral valve specimen from different angles, showing a greyish white-thickening and retraction of leaflets with small oval shaped orifice, commissural fusions with some calcification, and chordal thickening and shortening.

On gross examination, the excised MV showed extensive fibrotic thickening of the leaflets with commissural fusion, a rigid oval to slit like orifice and fibrotic retraction, and fusion of chordae up to the apical parts of the papillary muscles (*Figure 3*). Macroscopically, similarities with end-stage rheumatic MV disease were evident.

**Figure 4:**
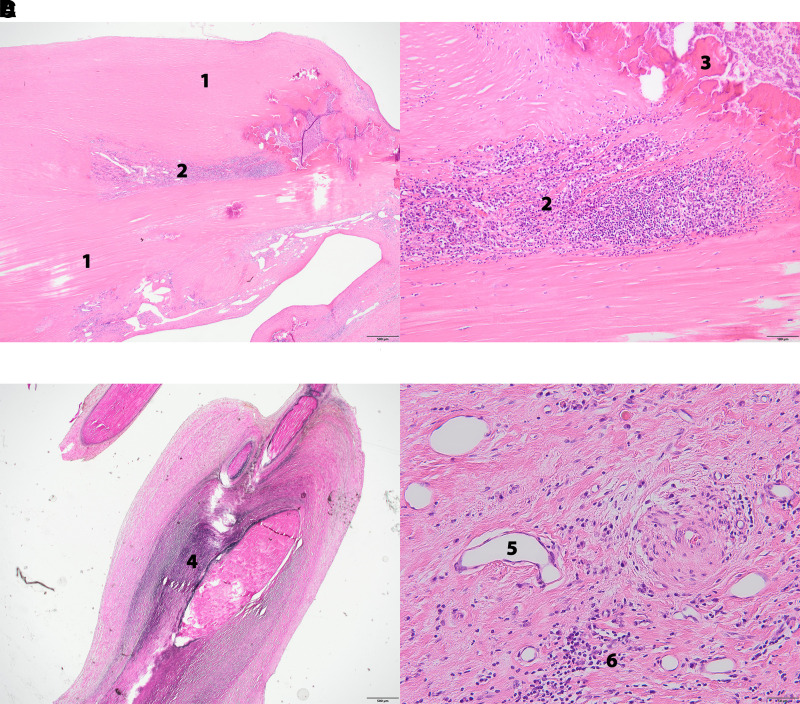
Histology of mitral valve. (*A*) Low power view of leaflet with dense collagenous tissue (1) and small focal inflammatory infiltrates (2) (haematoxylin and eosin stain, 40×). (*B*) Detail taken from (*A*), showing inflammatory infiltrate composed of mononuclear cells (2) and presence of calcification (3) in right upper corner (haematoxylin and eosin stain, 100×). (*C*) Section through thickened chorda surrounded by fibro elastic tissue (4) (EvG stain). (*D*) Detail of micro vessels (5) surrounded by inflammatory infiltrate (6), in which also plasma cells are present (haematoxylin and eosin stain, 200×).

Histologic analysis of MV leaflets and adjacent chordal tissues using haematoxylin and eosin stain and elastic von Gieson stain showed thickening of the valves due to dense collagen-rich fibrous tissue admixed with elastic fibres and scarce calcifications. Within the fibrous tissue, there was a multifocal presence of mononuclear cell infiltration composed of macrophages, T-lymphocytes and plasma cells, and neovascularization by dilated micro vessels and some thick-walled muscular vessels. Granulomas or necrosis was not observed. Also tendinous cords were surrounded by closely packed cords of fibro elastic connective tissue. Due to the high amount of plasma cells, IgG and IgG4 immunostains were performed, showing only small fraction of IgG4 positive cells, which excluded the presence of IgG4-related disease. For histology, see *Figure 4*.

Fragments of the resected AV were also examined histologically, and showed a basically similar histologic composition of fibrous thickening and scarce mononuclear inflammatory cell infiltration.

## Discussion

Ankylosing spondylitis is known to have cardiac involvement. Most occurring among these cardiac involvements is AV regurgitation and conduction disorders, in rare cases MV regurgitation has been described. ^2,3^ The suggested mechanism for the latter, is sub-aortic tissue fibrosis reaching MV leaflets causing decreased leaflet mobility.^10^ However, only scarce literature is available on this topic.

We report a case of a 51-year-old male with HLA-B27-negative AS suffering from heart valve disease. Main focus at eventual worsening of clinical complaints was MV stenosis. Surgical treatment was performed followed by histopathologic analysis of the excised MV. Histopathologic analysis showed chronic inflammation and fibrous thickening of the MV leaflets and subvalvular apparatus, which fits a diagnosis of ‘post-inflammatory valve disease’ characteristic for rheumatic heart disease. Since co-occurrence of other rheumatic diseases, particularly rheumatic fever and rheumatoid arthritis, was excluded in this patient, we postulate that the observed post-inflammatory valvular pathology relates to AS.

Though innate immune cells like macrophages were present within the fibrous tissue, the presence of T cells and plasma cells support an autoimmune rather than an autoinflammatory origin. Probably in our patient, a persistent low-grade chronic autoimmune induced inflammatory process has lead on the long term to the observed fibrous thickening and valvular deformation.

An arguable fact in this case is the HLA-B27 negative profile of the AS diagnosis. However, HLA-B27 is not a necessity in SpA diagnostics. In fact, 10% of patients with axial SpA have a HLA-B27 negative profile.^11^ In the absence of HLA-B27, patients should meet the criteria of sacroiliitis on imaging and one or more features of SpA, according to the Assessment of SpondyloArthritis International Society classification criteria for the diagnosis axial SpA.^12^ Our patient meets these criteria by showing symmetric sacroiliitis on both X-ray and MRI and is suffering from long-term inflammatory back pain and uveitis. This makes the diagnosis of AS in this patient valid and consultation of academic SpA experts confirmed the diagnosis of AS.

In conclusion, this is a rare case of AS-induced three valve disease, which appeared to be most prominent in the MV, with features of autoimmune mediated post-inflammatory valvular pathology, which is characteristic for rheumatic diseases. Since the literature on this topic is scarce, we propose that this notion could possibly create a base for better cardiac management in this specific patient group.

## Lead author biography


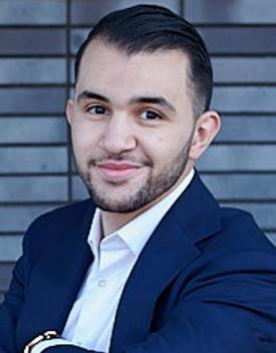
Sulayman el Mathari completed his study in medicine at the University of Amsterdam. Currently, he is a medical doctor and PhD candidate in the Department of Cardiothoracic Surgery at the Amsterdam University Medical Center.

## Supplementary Material

ytac322_Supplementary_Data
