# Supplementary material for: Mitral valve disease in ankylosing spondylitis: an autoimmune disease manifestation? A case report
Source: Eur Heart J Case Rep. 2022 Aug 1;6(8):ytac322. doi: 10.1093/ehjcr/ytac322 (PMC9443989; doi:10.1093/ehjcr/ytac322)
Supplement: ytac322_Supplementary_Data [file ytac322_Supplementary_Data.pptx]

## Slide 1
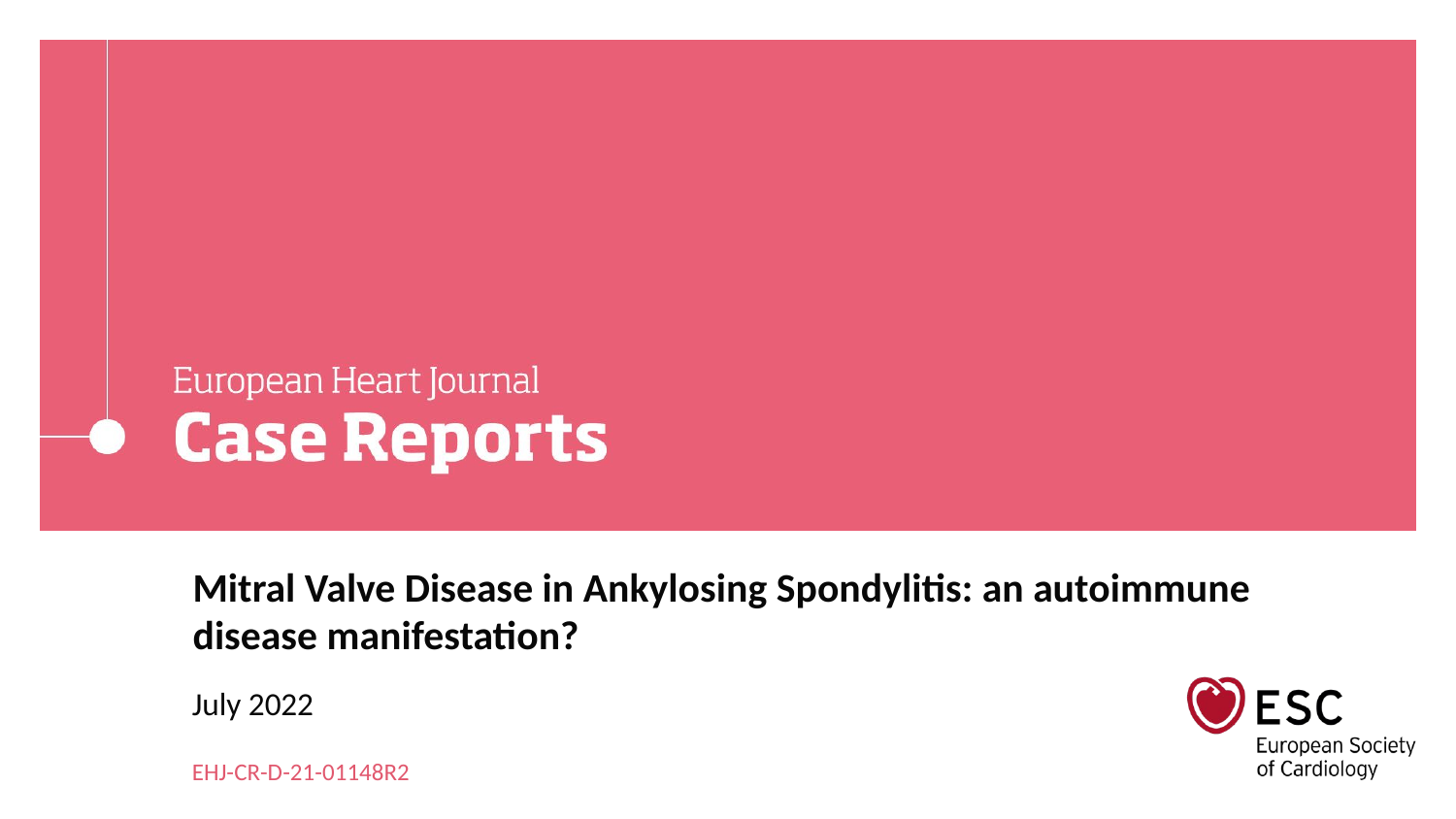

# Mitral Valve Disease in Ankylosing Spondylitis: an autoimmune disease manifestation?
July 2022
EHJ-CR-D-21-01148R2

## Slide 2
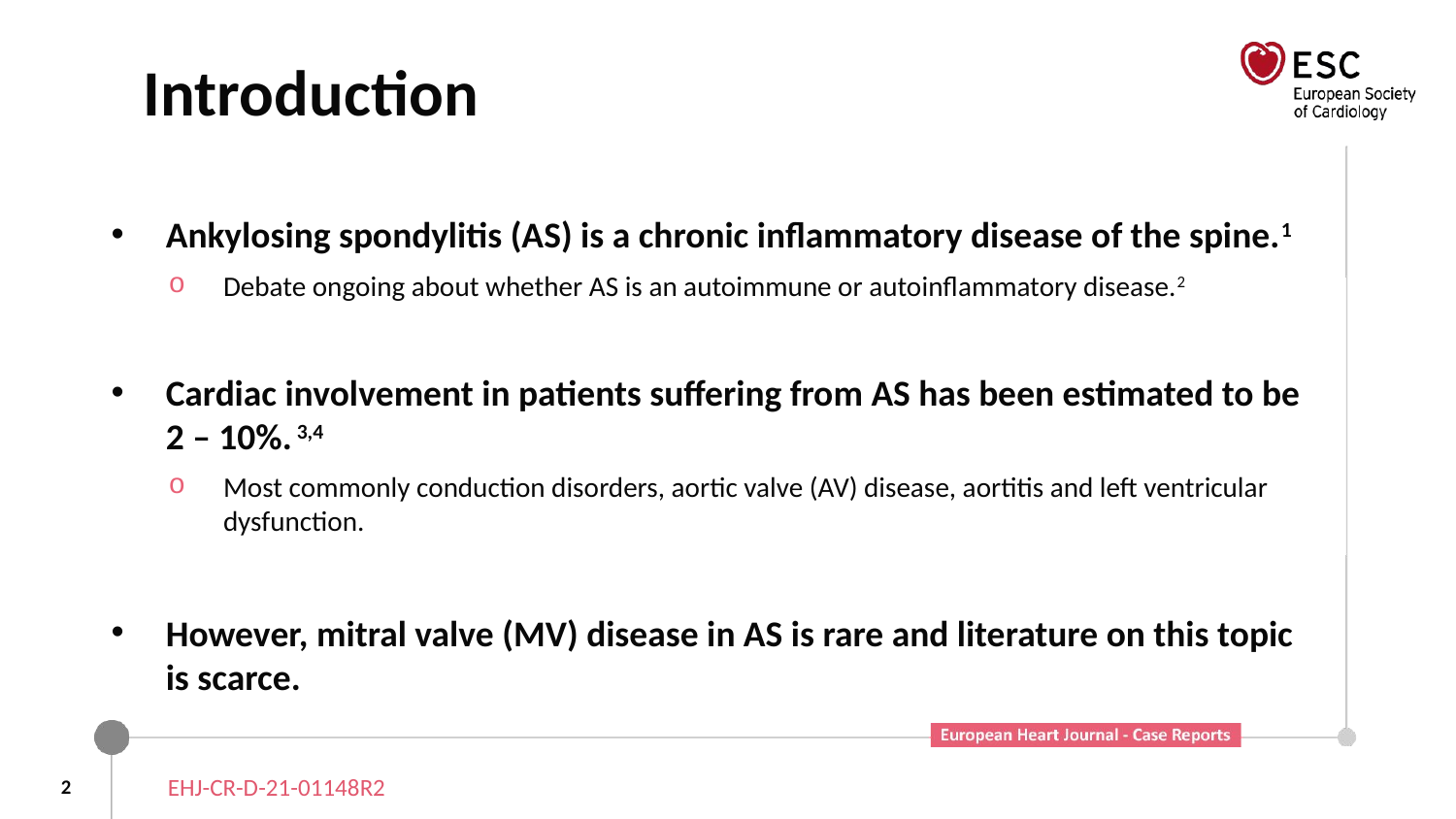

# Introduction
Ankylosing spondylitis (AS) is a chronic inflammatory disease of the spine.1
Debate ongoing about whether AS is an autoimmune or autoinflammatory disease.2
Cardiac involvement in patients suffering from AS has been estimated to be 2 – 10%. 3,4
Most commonly conduction disorders, aortic valve (AV) disease, aortitis and left ventricular dysfunction.
However, mitral valve (MV) disease in AS is rare and literature on this topic is scarce.
2
EHJ-CR-D-21-01148R2

## Slide 3
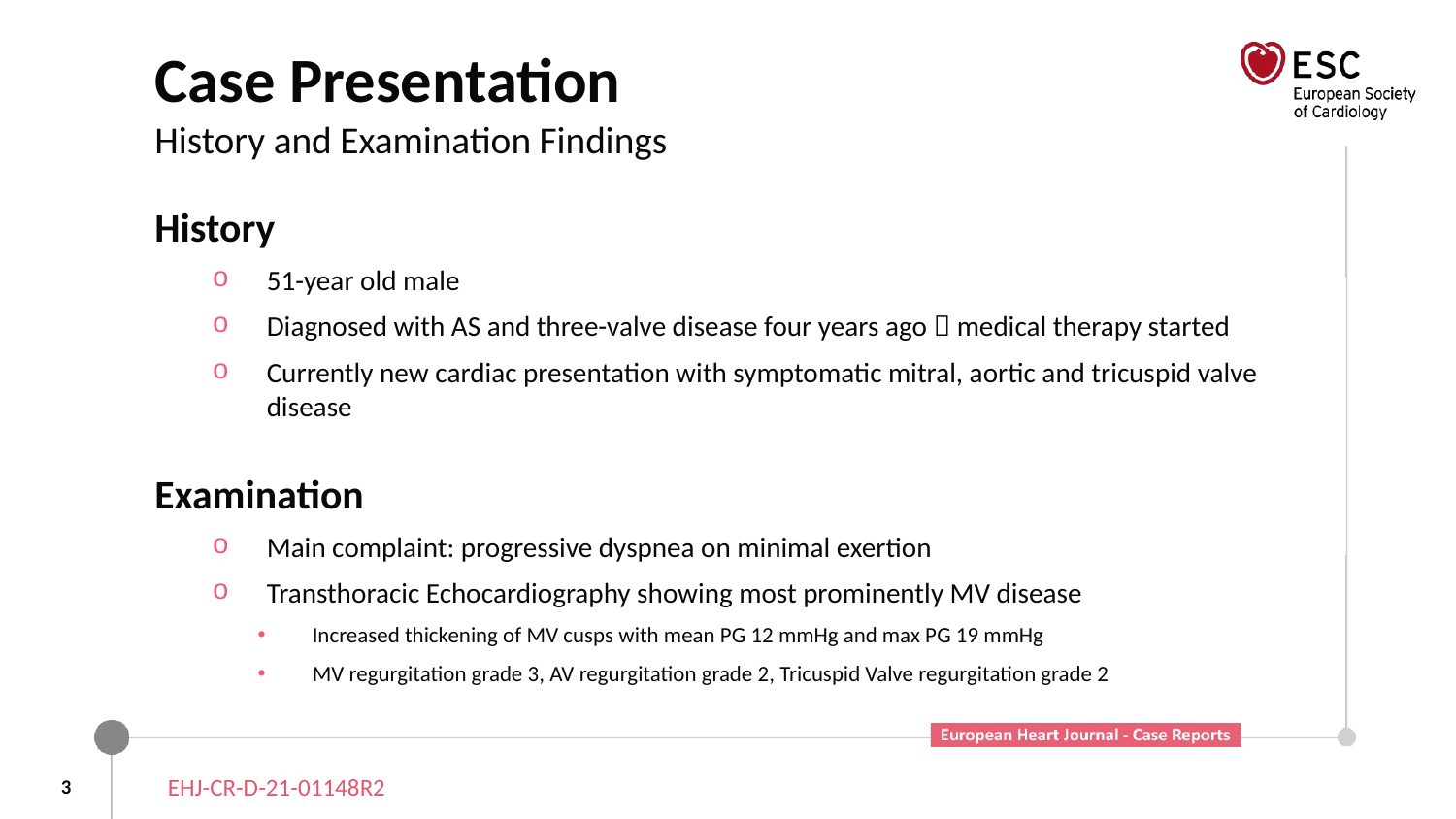

# Case Presentation History and Examination Findings
History
51-year old male
Diagnosed with AS and three-valve disease four years ago  medical therapy started
Currently new cardiac presentation with symptomatic mitral, aortic and tricuspid valve disease
Examination
Main complaint: progressive dyspnea on minimal exertion
Transthoracic Echocardiography showing most prominently MV disease
Increased thickening of MV cusps with mean PG 12 mmHg and max PG 19 mmHg
MV regurgitation grade 3, AV regurgitation grade 2, Tricuspid Valve regurgitation grade 2
3
EHJ-CR-D-21-01148R2

## Slide 4
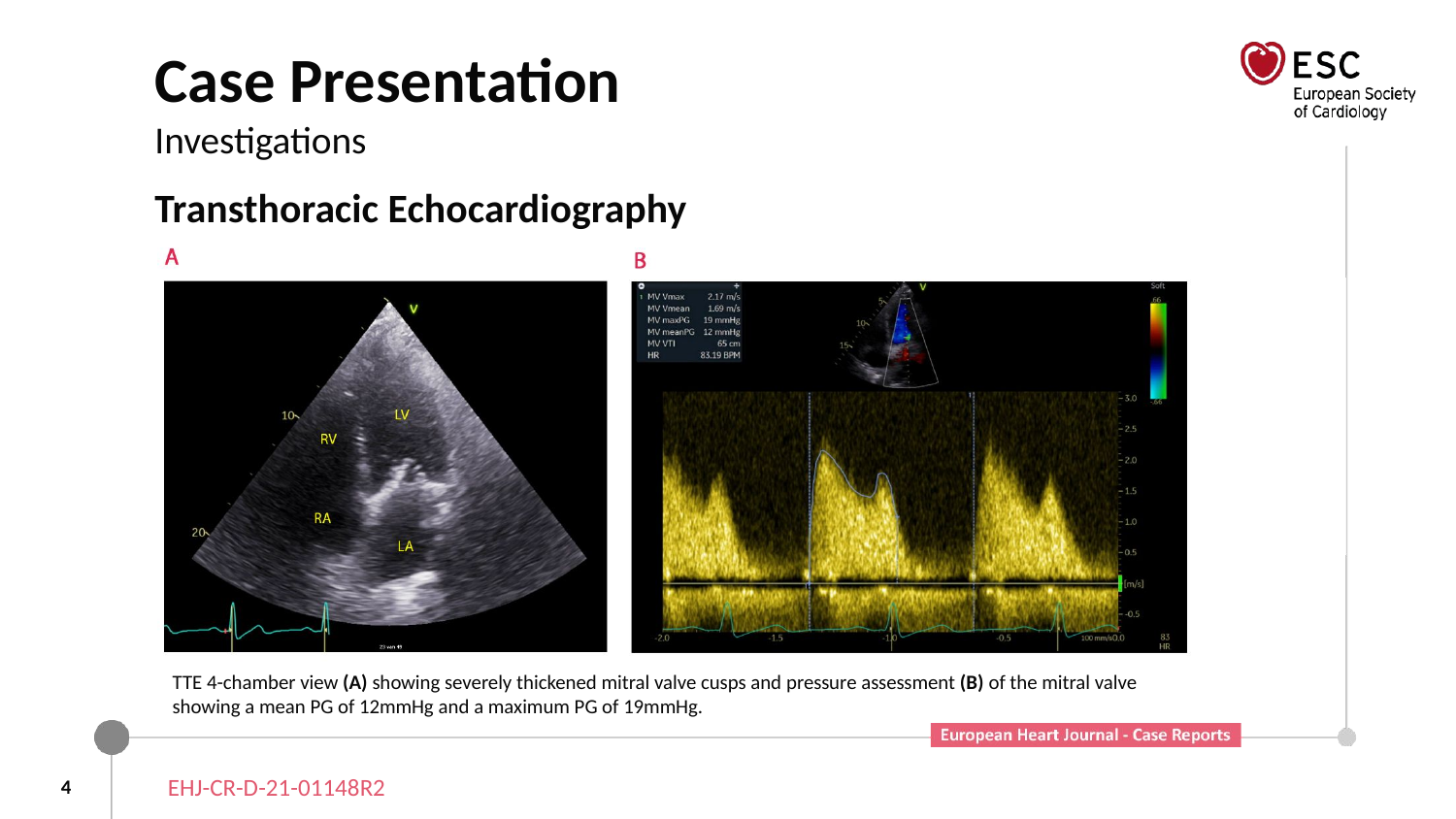

# Case Presentation Investigations
Transthoracic Echocardiography
TTE 4-chamber view (A) showing severely thickened mitral valve cusps and pressure assessment (B) of the mitral valve showing a mean PG of 12mmHg and a maximum PG of 19mmHg.
4
EHJ-CR-D-21-01148R2

## Slide 5
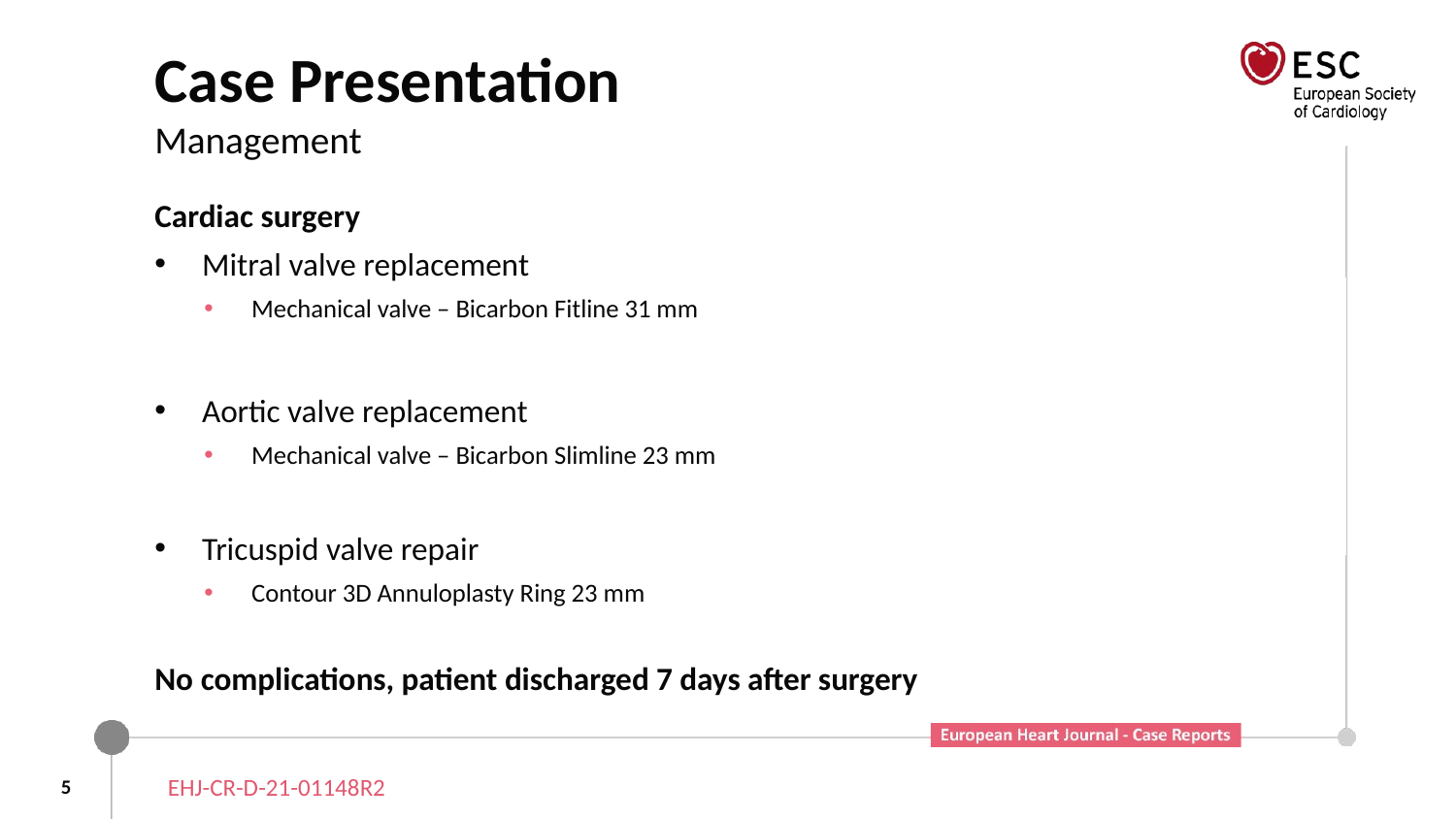

# Case Presentation Management
Cardiac surgery
Mitral valve replacement
Mechanical valve – Bicarbon Fitline 31 mm
Aortic valve replacement
Mechanical valve – Bicarbon Slimline 23 mm
Tricuspid valve repair
Contour 3D Annuloplasty Ring 23 mm
No complications, patient discharged 7 days after surgery
5
EHJ-CR-D-21-01148R2

## Slide 6
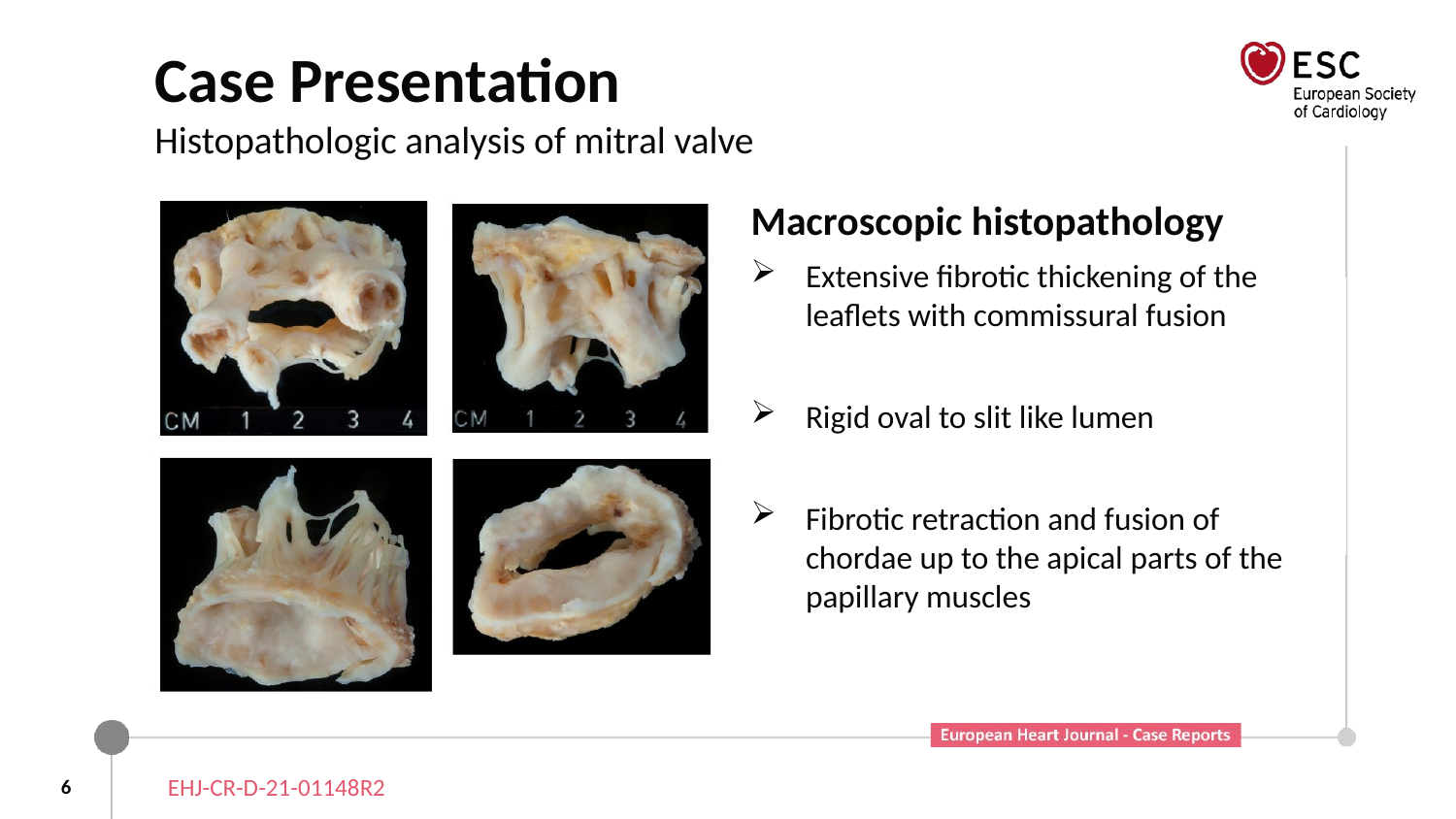

# Case Presentation Histopathologic analysis of mitral valve
Macroscopic histopathology
Extensive fibrotic thickening of the leaflets with commissural fusion
Rigid oval to slit like lumen
Fibrotic retraction and fusion of chordae up to the apical parts of the papillary muscles
6
EHJ-CR-D-21-01148R2

## Slide 7
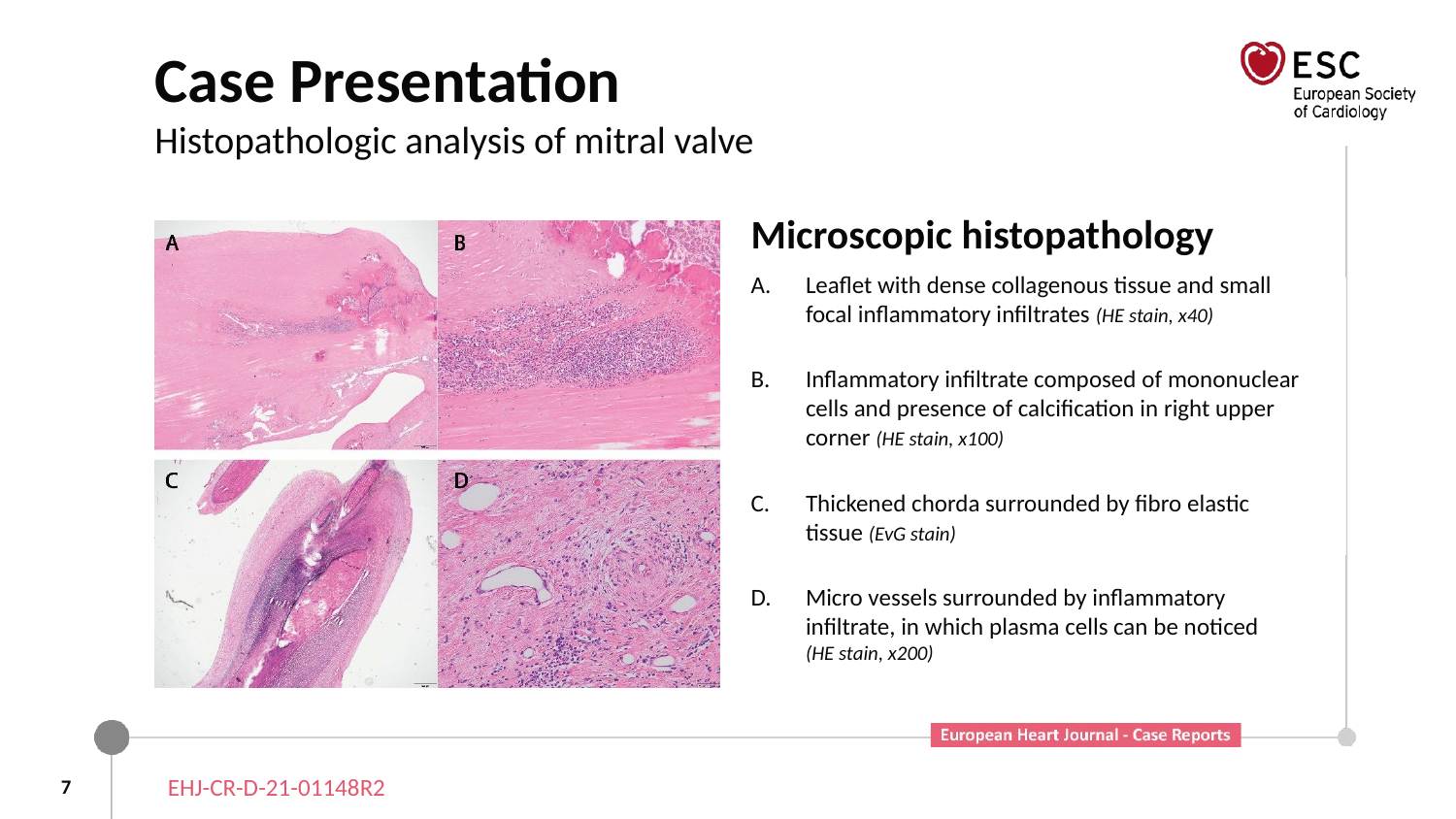

# Case Presentation Histopathologic analysis of mitral valve
Microscopic histopathology
Leaflet with dense collagenous tissue and small focal inflammatory infiltrates (HE stain, x40)
Inflammatory infiltrate composed of mononuclear cells and presence of calcification in right upper corner (HE stain, x100)
Thickened chorda surrounded by fibro elastic tissue (EvG stain)
Micro vessels surrounded by inflammatory infiltrate, in which plasma cells can be noticed
(HE stain, x200)
7
EHJ-CR-D-21-01148R2

## Slide 8
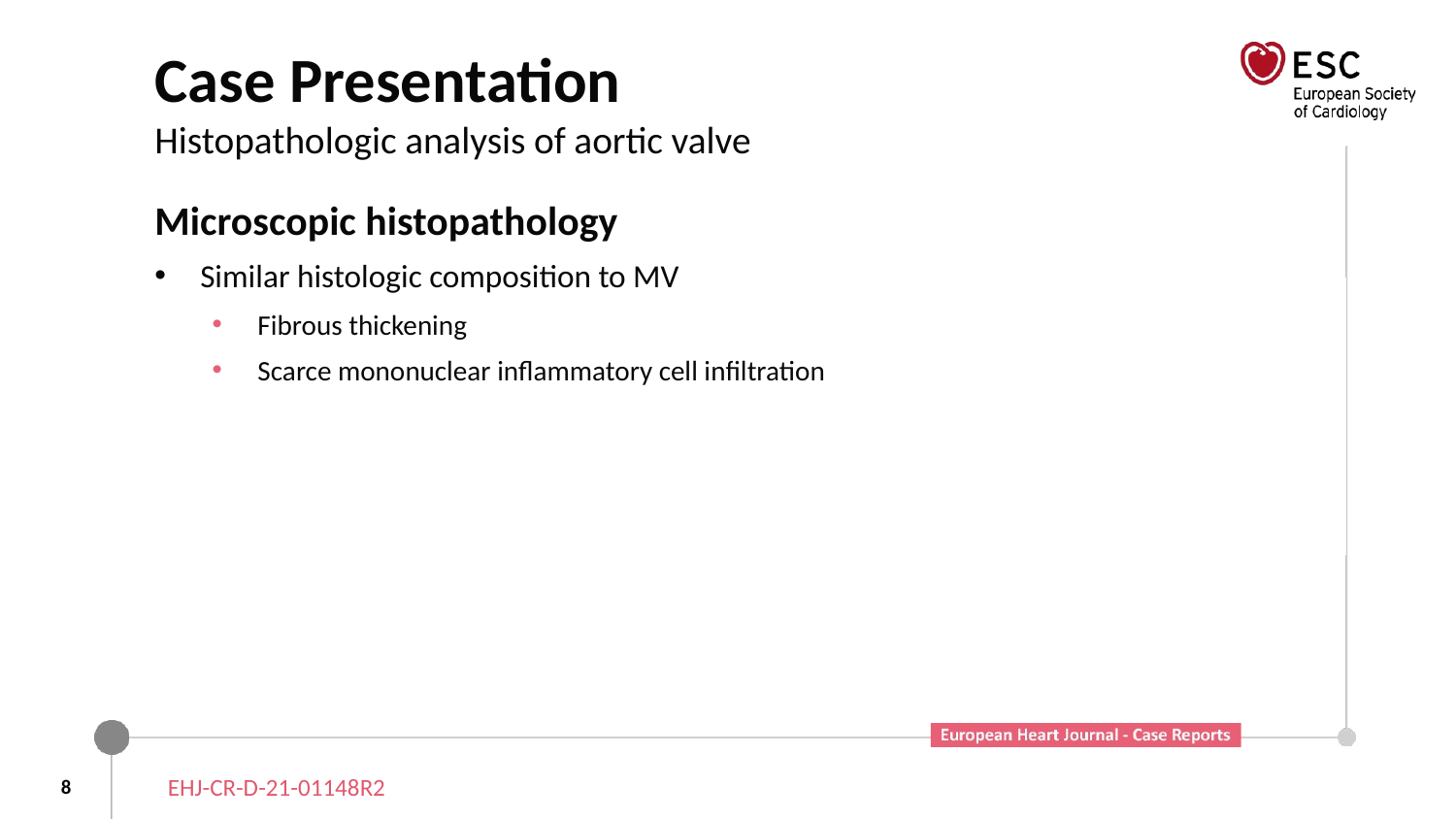

# Case Presentation Histopathologic analysis of aortic valve
Microscopic histopathology
Similar histologic composition to MV
Fibrous thickening
Scarce mononuclear inflammatory cell infiltration
8
EHJ-CR-D-21-01148R2

## Slide 9
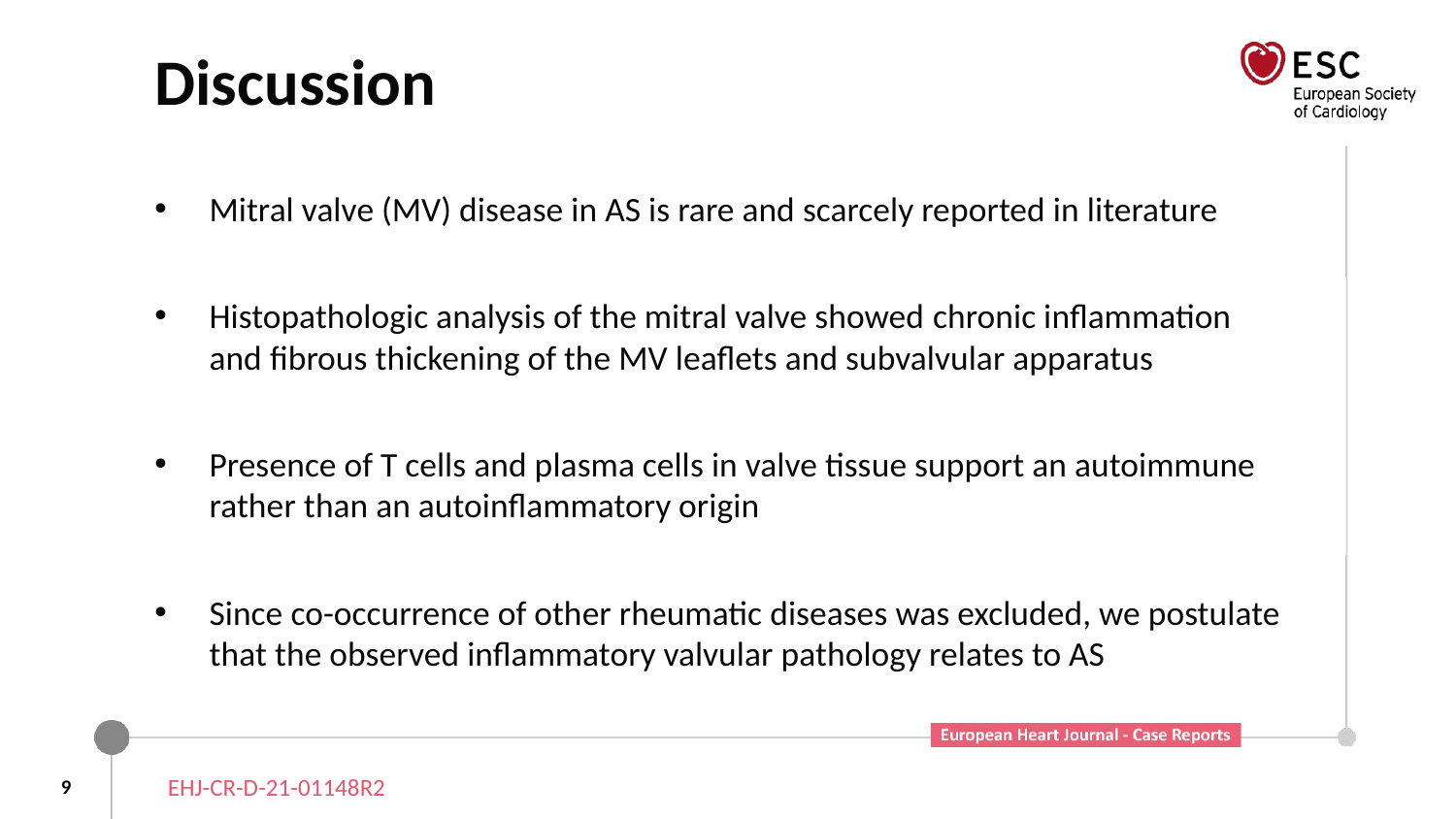

# Discussion
Mitral valve (MV) disease in AS is rare and scarcely reported in literature
Histopathologic analysis of the mitral valve showed chronic inflammation and fibrous thickening of the MV leaflets and subvalvular apparatus
Presence of T cells and plasma cells in valve tissue support an autoimmune rather than an autoinflammatory origin
Since co-occurrence of other rheumatic diseases was excluded, we postulate that the observed inflammatory valvular pathology relates to AS
9
EHJ-CR-D-21-01148R2

## Slide 10
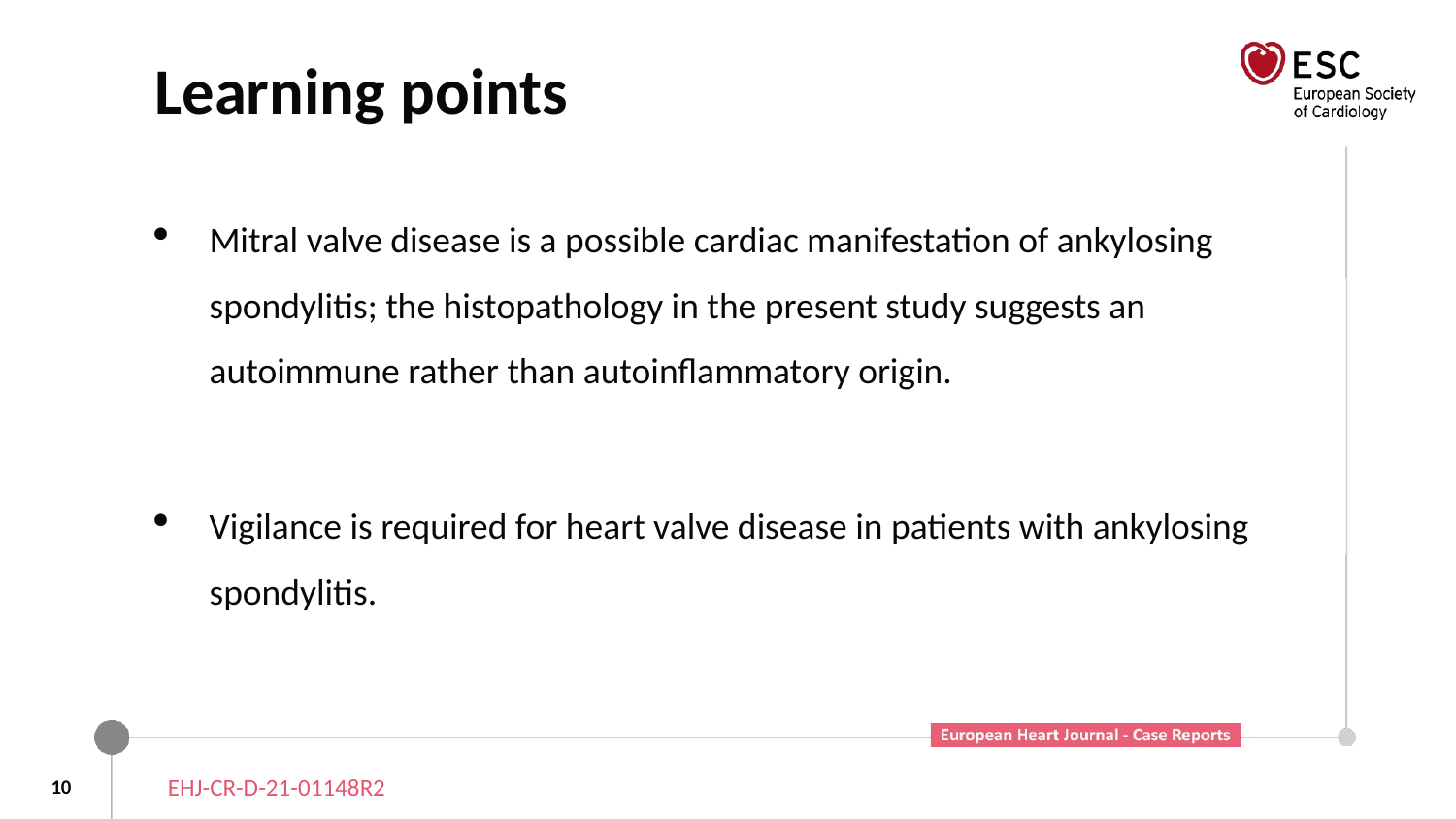

# Learning points
Mitral valve disease is a possible cardiac manifestation of ankylosing spondylitis; the histopathology in the present study suggests an autoimmune rather than autoinflammatory origin.
Vigilance is required for heart valve disease in patients with ankylosing spondylitis.
10
EHJ-CR-D-21-01148R2

## Slide 11
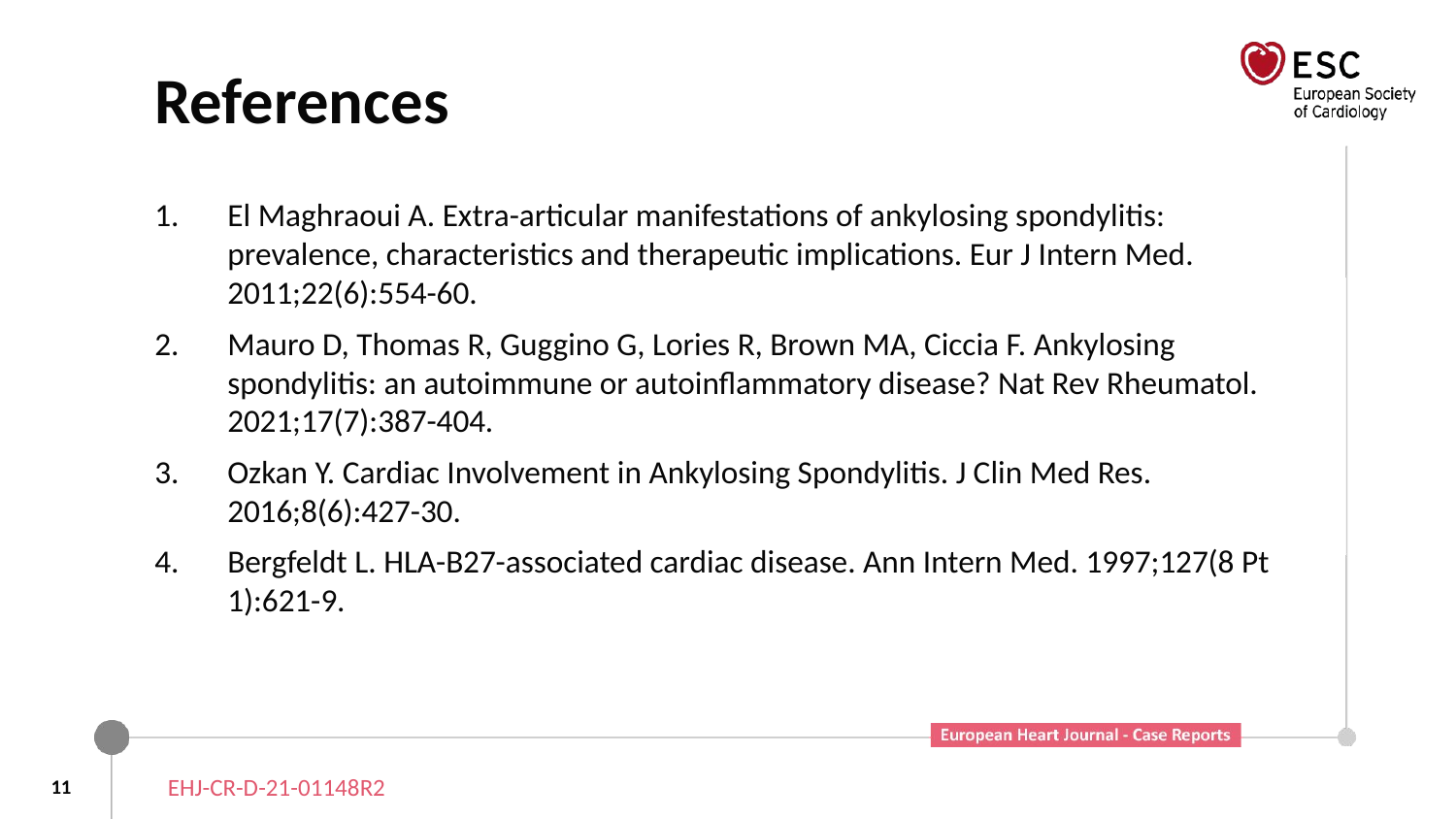

# References
El Maghraoui A. Extra-articular manifestations of ankylosing spondylitis: prevalence, characteristics and therapeutic implications. Eur J Intern Med. 2011;22(6):554-60.
Mauro D, Thomas R, Guggino G, Lories R, Brown MA, Ciccia F. Ankylosing spondylitis: an autoimmune or autoinflammatory disease? Nat Rev Rheumatol. 2021;17(7):387-404.
Ozkan Y. Cardiac Involvement in Ankylosing Spondylitis. J Clin Med Res. 2016;8(6):427-30.
Bergfeldt L. HLA-B27-associated cardiac disease. Ann Intern Med. 1997;127(8 Pt 1):621-9.
11
EHJ-CR-D-21-01148R2
